# Oxalate-induced apoptosis through ERS-ROS–NF-κB signalling pathway in renal tubular epithelial cell

**DOI:** 10.1186/s10020-022-00494-5

**Published:** 2022-08-03

**Authors:** Shaoxiong Ming, Jia Tian, Ke Ma, Chengbin Pei, Ling Li, Zeyu Wang, Ziyu Fang, Min Liu, Hao Dong, Weijian Li, Jianwen Zeng, Yonghan Peng, Xiaofeng Gao

**Affiliations:** 1 Department of Urology, Changhai Hospital of Shanghai, No. 168, Changhai Road, Yangpu District, Shanghai, 200433 China; 2 Department of Human Sperm Bank of Ningxia, General Hospital of Ningxia Medical University, Ningxia Medical University, No. 804 Shengli South Street, Xingqing District, Yinchuan, 750001 Ningxia Hui Autonomous Region China; 3 Department of Urology, Sixth Affiliated Hospital of Guangzhou Medical University (Qingyuan People’s Hospital), B24, Yinquan Road, XinchengDistrict, Qingyuan, 511518 Guangdong Province China

**Keywords:** Kidney stones, Oxalate, Endoplasmic reticulum stress (ERS), Reactive oxygen species (ROS), NF-κB signalling pathway

## Abstract

**Background:**

Kidney stones are composed of approximately 70–80% calcium oxalate. However, the exact mechanism of formation of calcium oxalate kidney stones remains unclear. In this study, we investigated the roles of endoplasmic reticulum stress (ERS), reactive oxygen species (ROS), and the NF-κB signalling pathway in the pathogenesis of oxalate-induced renal tubular epithelial cell injury and its possible molecular mechanisms.

**Methods:**

We established a model to evaluate the formation of kidney stones by intraperitoneal injection of glyoxylic acid solution into mice and assessed cell morphology, apoptosis, and the expression levels of ERS, ROS, and NF-κB signalling pathway-related proteins in mouse renal tissues. Next, we treated HK-2 cells with potassium oxalate to construct a renal tubular epithelial cell injury model. We detected the changes in autophagy, apoptosis, and mitochondrial membrane potential and investigated the ultrastructure of the cells by transmission electron microscopy. Western blotting revealed the expression levels of apoptosis and autophagy proteins; mitochondrial structural and functional proteins; and ERS, ROS, and NF-κB (p65) proteins. Lastly, we studied the downregulation of NF-κB activity in HK-2 cells by lentivirus interference and confirmed the interaction between the NF-κB signalling and ERS/ROS pathways.

**Results:**

We observed swelling of renal tissues, increased apoptosis of renal tubular epithelial cells, and activation of the ERS, ROS, and NF-κB signalling pathways in the oxalate group. We found that oxalate induced autophagy, apoptosis, and mitochondrial damage in HK-2 cells and activated the ERS/ROS/NF-κB pathways. Interestingly, when the NF-κB signalling pathway was inhibited, the ERS/ROS pathway was also inhibited.

**Conclusion:**

Oxalate induces HK-2 cell injury through the interaction between the NF-κB signalling and ERS/ROS pathways.

## Background

Kidney stones are common in the urinary system, and often adversely affect human health. The annual incidence of kidney stones has increased by 14.8%, and recurrence within 5 years of initial diagnosis is as high as 50% (Zisman 2017; Khan et al. 2016). Approximately 70–80% of kidney stones comprise calcium oxalate, and the proportion of calcium oxalate is greater than 70%, even in heterogenous stones (Khan et al. 2016). Currently, the exact mechanism by which calcium oxalate kidney stones form is unclear. Hyperoxaluria and crystal deposition are closely related to lipid oxidation and renal tubular epithelial cell injury (Mittal et al. 2016). Therefore, it is important to investigate the pathogenesis of calcium oxalate kidney stones, and to devise targeted preventive measures to prevent the recurrence of such stones and reduce the consequent economic burden.

The endoplasmic reticulum (ER) is the most important intracellular chamber involved in the secretory pathway of eukaryotic cells (Loi et al. 2021). Various organelles with important physiological functions participate in the synthesis and folding of proteins, and the storage and release of calcium. Furthermore, a series of processes including lipid and steroid metabolism play important roles in maintaining the stable synthesis of Ca^2+^ and proteins in cells (Oakes 2020). Stress stimuli—such as ischaemia, hypoxia, lack of nutrition, and the production of numerous free radicals—can destroy ER homeostasis and lead to the accumulation of misfolded/folded proteins in the ER lumen. This triggers ER stress (ERS). Moderate ERS is beneficial for the recovery of intracellular calcium and the homeostasis of protein processing. It also enhances the ability of cells to withstand stress stimulation. However, if ERS persists or is too strong, the cell enters apoptosis.

**Fig.1:**
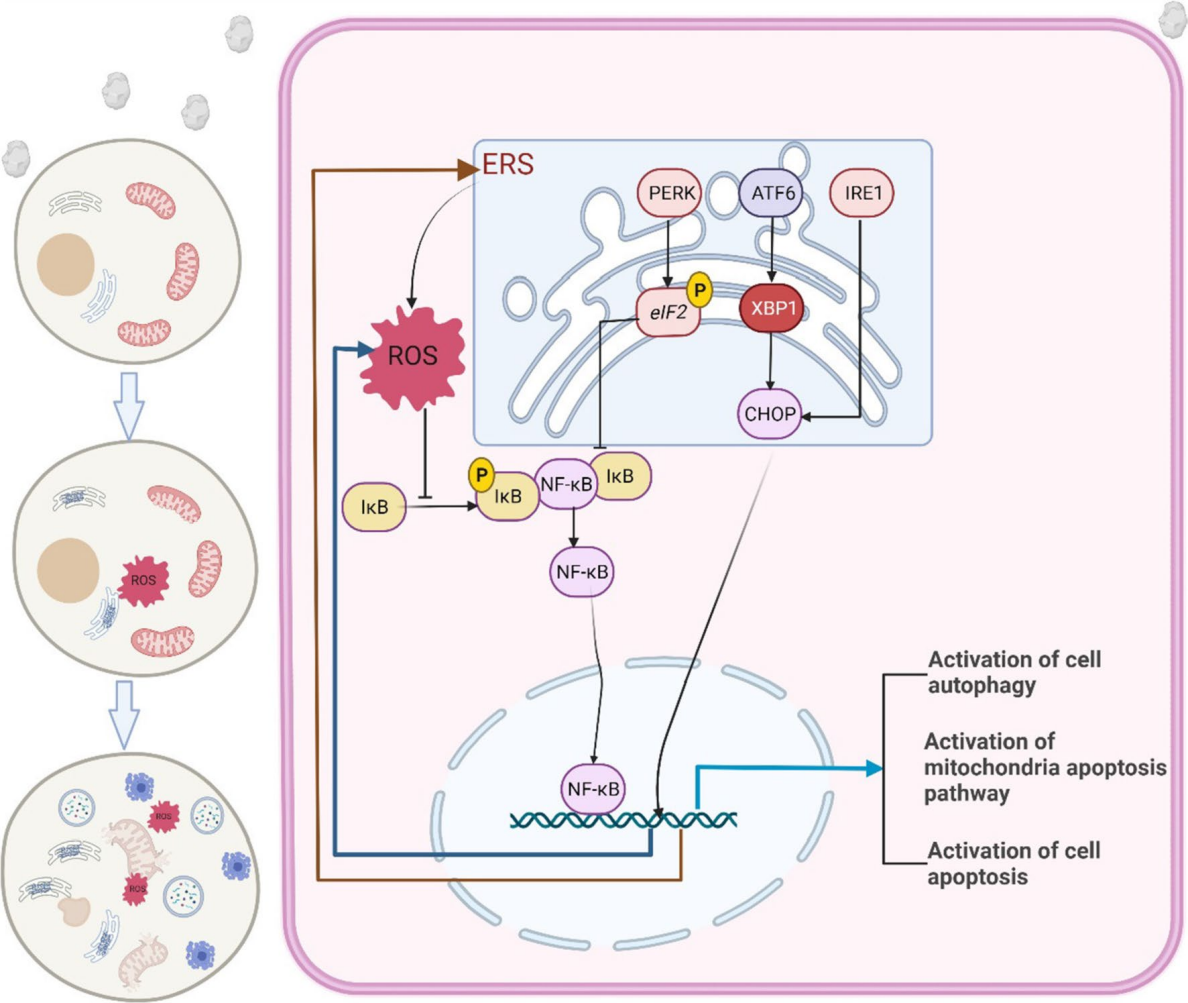
Oxalate induces renal tubular epithelial cell injury by activating ERS/ROS/NF-κB signaling pathway

Nuclear factor kappa-light-chain-enhancer of activated B cells (NF-κB) is an important nuclear transcription factor. It plays a key role in the regulation of cellular information transcription mediated by various cell stimuli; regulates the expression of genes related to cell survival, proliferation, and differentiation; and plays an important role in cell apoptosis. Moreover, it is considered to constitute a classic inflammation-related signalling pathway (Zhang et al. 2017). In this study, we found that oxalate activates the generation of ROS and the NF-κB signalling pathway by causing ERS, inducing mitochondrial dysfunction in HK-2 cells, enhancing autophagy, and promoting apoptosis (Fig. 1).

## Methods

### Main reagents and consumables

The kits for detecting apoptosis (KeyGEN Biotech, KGA107) and mitochondrial membrane potential (KeyGEN Biotech, KGA603) were purchased from Nanjing KGI Bio. The NF-κB interference lentivirus and autophagy lentivirus were purchased from Shanghai GeneChem Co., Ltd. Potassium oxalate (6487-48-5) solution was purchased from the Rhawn Reagent Co., Ltd. Glyoxylic acid solution (298-12-4) was purchased from the Aladdin Company. Antibody-related information is provided in Supplementary 1.

### Animals and groups

We placed C57BL/6 mice (n = 30, male, 6 weeks old, 20–25 g body weight) in a controlled environment comprising a light cycle of 12 h, a temperature of 20–25 °C, and a humidity of 45–55%. The mice were allowed ad libitum access to food and water. They were randomly divided into a control group (n = 10), an oxalate group (n = 10), and an inhibitor group (n = 10).

### Kidney stone mouse model

The oxalate group was injected intraperitoneally with 0.1 mg/g glyoxylic acid solution once per day for 5 days to establish a kidney stone mouse model (Ye et al. 2021a; Lu et al. 2021). The inhibitor group was injected intraperitoneally with the ERS inhibitor tauroursodeoxycholic acid (0.5 g/kg) for 24 h, then injected intraperitoneally with 0.1 mg/g glyoxylic acid solution once per day for 5 days. The normal group received the same amount of normal saline injected intraperitoneally once per day for 5 consecutive days. The mice were euthanised and decapitated 48 h after the last gavage, and renal tissue was collected for detection.

### Observation of renal histomorphology

The kidney tissue was fixed with 40 g/L neutral formaldehyde solution for 24 h and embedded in paraffin. Sections of the embedded tissue were stained with haematoxylin and eosin (HE), and the tissue morphology was examined under an optical microscope. We investigated histological changes in the kidney with a microscope and severe lesions with a 400× high-power microscope; we selected 100 renal tubules from 10 high-power microscope fields according to the method described by Lv et al. (2021) In the method described, two histopathologists scored the renal damage grades, randomly selected different visual fields, and calculated the proportion of renal tubular damage. Injury was scored as 1 for 5 renal tubular damage, 1 for ≤ 10%, 2 for 11–25%, 3 for 26–45%, 4 for 46–75%, and 5 for ≥ 76%.

### Terminal deoxynucleotidyl transferase-mediated nick end labelling (TUNEL) staining for renal tissue apoptosis detection

Renal tissue sections (5 μm thick) were prepared by paraffin embedding. After dewaxing with xylene and hydration with an ethanol gradient, the sections were washed three times with phosphate-buffered saline (PBS) for 5 min each time. The excess liquid around the tissue on the glass slide was carefully absorbed with filter paper. Next, the TUNEL detection kit working solution was adjusted according to the operation flow of the kit and added dropwise to the sample on the slide, which was then incubated at 37 °C for 1 h, washed three times with PBS, stained with 4′,6-diamidino-2-phenylindole (DAPI) solution at 37 °C for 30 min, and washed three more times with PBS. The contents of the sealed tablets from the kit were then added dropwise. The cells were examined and photographed using a fluorescence microscope.

### ELISA detection

The detection kits for superoxide dismutase (SOD), catalase (CAT), malondialdehyde (MDA), and glutathione (GSH) were purchased from Nanjing Jiancheng Bioengineering Institute (Nanjing, China). The kidney tissues of mice in each group were ground on ice using an electric tissue grinder for 35 min. The cell suspension was centrifuged for 10 min at 12,000 rpm. The supernatant was transferred to a new 1.5 mL centrifuge tube. Protein concentration was determined by the Bicinchoninic Acid (BCA) method performed thrice.

### Cell culture

Human renal cortex proximal convoluted tubule epithelial cells (HK-2 cells) in minimum essential medium containing 10% foetal bovine serum (Gibco, 10091148), 100 U/L penicillin, and 0.1 g/L streptomycin (Solarbio, P1400) for cell culture were purchased from Gibco. Logarithmic-phase cells exhibiting good growth were used in the experiment.

### Cell experiment grouping

HK-2 cells with normal growth were used as the control group. After 24 h, we simultaneously added tauroursodeoxycholic acid (the stress inhibitor; 200 μm) to the cells of the inhibitor group and potassium oxalate (5 mmol/L) to those of the oxalate group. After 72 h, the cells from each group were collected for subsequent detection.

### Detection of apoptosis by flow cytometry

We digested the cells in 0.25% pancreatin, washed them three times in PBS, counted the cells, collected 10^6^ cells per sample, and repeated the procedure to obtain three samples from each group. We then added binding buffer (100 μL) to resuspend the cells, transferred them to a flow tube, added Annexin V-FITC (5 μL) and propidium iodide dye solution (5 μL), and incubated the cells at room temperature for 30 min. Finally, we added binding buffer (400 μL), mixed the cells thoroughly, and detected fluorescence using a flow cytometer (FACSVantage™ SE, BD Biosciences, Canada). The data were processed using CFlow software.

### TUNEL staining to detect apoptosis in the HK-2 cells

We prepared cell sections from each group, washed them three times with PBS, fixed them with 75% ethanol for 30 min, washed them three times with PBS, dripped the membrane-breaking working solution onto the slides so that it covered the tissues, incubated the samples at room temperature for 20 min, and washed them three more times with PBS. We adjusted the TUNEL detection working solution according to the operation flow of the kit, dripped the TUNEL solution onto the samples, incubated the samples at 37 °C for 1 h, washed them three times with PBS, dyed them with DAPI staining solution at room temperature for 30 min, and washed them three times with PBS. The contents of the sealed tablets from the kit were then added dropwise.

### Determination of the mitochondrial membrane potential (MMP) by flow cytometry

We digested the cells in 0.25% pancreatin, washed them three times with PBS, counted the cells, collected 10^6^ cells per sample, and repeated the procedure to obtain three samples from each group. Next, we prepared a JC-1 probe dye solution according to the manufacturer’s instructions, incubated the cells in it at 37 °C for 30 min, washed the cells three times in PBS, and detected the MMP of each group by flow cytometry.

### Determination of the MMP by fluorescence staining

The cell culture solutions were removed from the 6-well plates used for each of the three groups of cells described above, the cells were washed twice, and 1 mL of the cell culture solution and 1 mL of the JC-1 staining solution were added. The cells were mixed thoroughly. After incubation at 37 °C for 20 min, the supernatant was aspirated and the cells were washed twice with JC-1 staining buffer (1X). Then, 2 mL/well of the cell culture solution was added. The cells were examined under a fluorescence microscope. The excitation light was set at 490 nm and the emission light was set at 530 nm to detect the JC-1 monomer. The excitation and emission wavelengths of the JC-1 polymer are 525 and 590 nm, respectively.

### Detection of the expression of related proteins by western blotting

We digested the cells in 0.25% pancreatin and washed them three times with PBS. The proteins in each group were extracted using a kit (KeyGEN Biotech, KGP2100), and the protein concentrations were measured using the bicinchoninic acid method. Sodium dodecyl sulphate–polyacrylamide gel electrophoresis was performed after protein quantification. Once the proteins had been transferred to a polyvinylidene fluoride membrane, they were sealed with 5% skim milk powder at room temperature for 1 h, and the corresponding antibodies were added. A gel imaging system was used for exposure, and the grey values of the images were analysed using Image Lab software. Colour was developed with an ECL developer and photographed using a gel imaging system (ChemiDoc XRS+, Bio-Rad, Canada). Image J v1.46r software was used to quantitatively analyse the protein expression.

### Examination of mitochondrial autophagosomes by transmission electron microscopy

The cells were digested in 0.25% trypsin, washed three times with PBS, fixed in glutaraldehyde solution, rinsed with PBS, dehydrated with 50% and 70% ethanol followed by an acetone gradient, embedded in an epoxy resin, sliced into 60 nm sections, mounted on a copper mesh, stained with saturated uranium acetate, washed with water, dipped in lead dye solution, washed with water, dried, and photographed under an electron microscope.

### Determination of ROS by fluorescence microscopy

We prepared the cell sections from each group, washed them three times with PBS, added with a final concentration of 10 μmol/L DCFH-DA (6-carboxy-2′,7′-dichlofluorescein diacetate), incubated in an incubator for 30 min, and rinsed thrice with PBS. Finally, photos were obtained under a fluorescence microscope.

### Determination of ROS using flow cytometer

The treatment of HK-2 cells was the same as above. The culture medium was discarded, and cells were added with a final concentration of 10 μmol/L DCFH-DA (6-carboxy-2′,7′-dichlorofluorescein diacetate) and incubated in an incubator for 30 min. The medium was discarded, and digestion with 0.25% pancreatin was performed. The cell suspension was transferred to a 1.5 mL centrifuge tube, washed thrice with PBS for 3 to fully remove excess DCFH-DA. A total of 100 μL PBS was added to resuspended cells. Fluorescence was detected using a flow cytometer (FACSVantage ™ SE, BD Biosciences, Canada), and data were processed using CFlow software.

### Statistical methods

We used the SPSS 21.0 software for statistical analyses, and GraphPad Prism 8 for drawing. Each item of data is reported as the mean and standard deviation ($\overline{x }\pm s$). We used variance analysis for data from multiple groups, and the standard *t*-test for comparisons between two groups. *P* < 0.05 indicates a statistically significant difference.

## Results

### Oxalate-induced kidney injury in kidney stone model mice

**Fig. 2:**
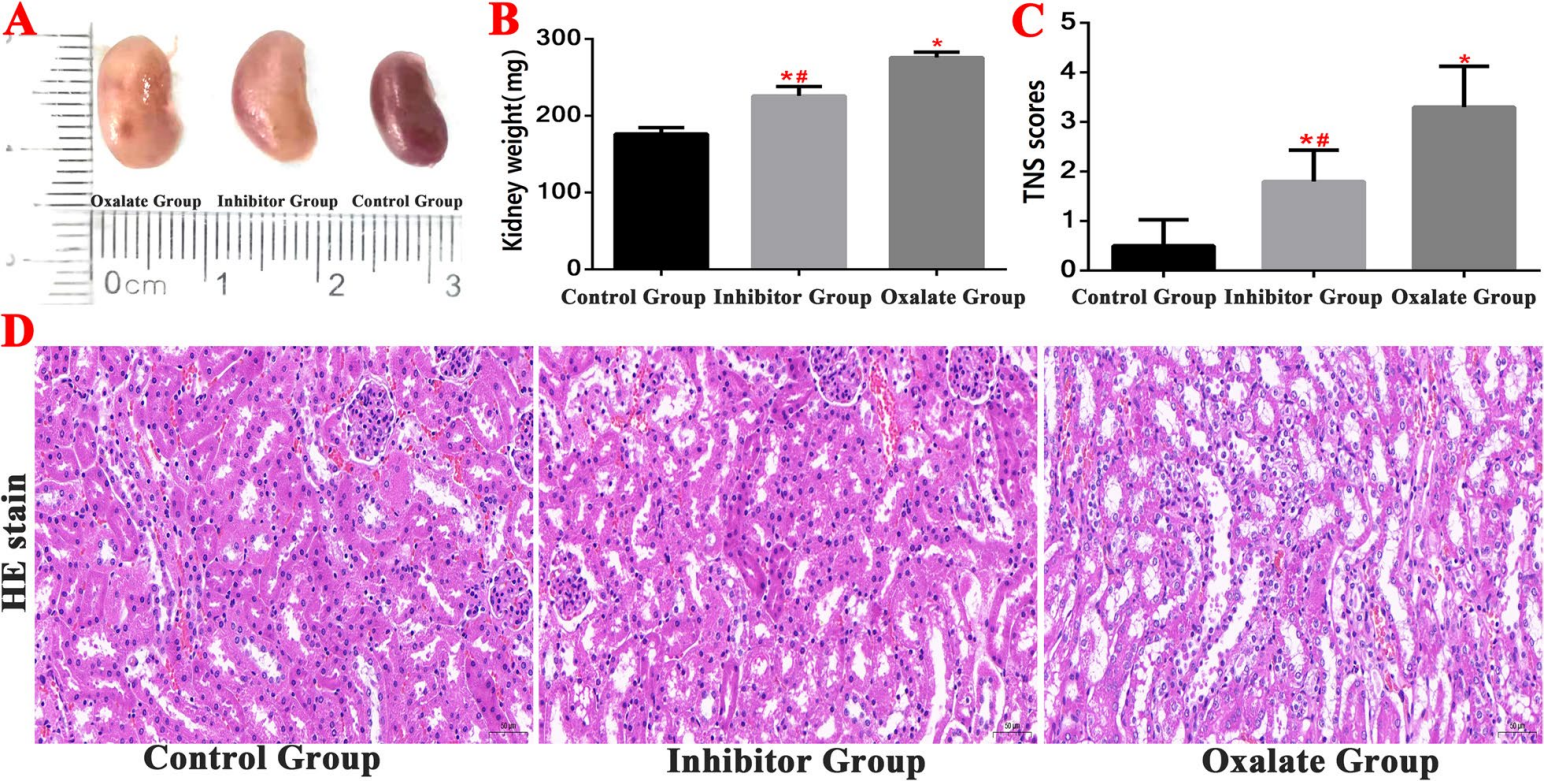
Detection of kidney tissue damage in Kidney stone model. **A**, **B** Comparison of kidney weight detection of mice in each group. **C**, **D** HE staining was used to observe the kidney structure and compare the renal tubular injury scores of mice in each group. (*^(Red)^Compared with control group, *P* < 0.01. ^#(Red)^Compared with oxalate group, *P* < 0.01)

During the modelling period, there was no death in each group. We monitored the kidneys of the mice in the normal, inhibitor, and oxalate groups. In the oxalate group, the kidney volume increased, the kidney tissue swelled, the renal capsule tightened, the kidney weight increased significantly, and the renal damage grade scores were higher than those of the control group (Fig. 2).

Haematoxylin and eosin staining and examination under a visible light microscope revealed that in the normal (control) group, the wall of the proximal tubule was thick, the lumen was small and irregular, the epithelial cells were cubic or conical, the cell body was large, the boundary was unclear, the lumen surface had a brush edge, and the nucleus, which was located near the basal part, was spherical. The distal tubule had a thin wall, a large and regular lumen, cuboid epithelial cells, a small cell body, a clear boundary, no brush edge on the free surface, and a spherical nucleus, which was located in the centre. The oxalate group exhibited obvious pathological changes: a deformed renal capsule and a narrowed or absent renal capsule cavity. The proximal tubules also exhibited obvious changes: swollen epithelial cells, numerous vacuoles in the cells, no brush edge, and necrotic cell fragments and interstitial oedema in the tubules. Compared to the oxalate group, there were significantly fewer changes to the renal tissue in the inhibitor group: the renal tubular epithelial cells were slightly oedematous, there were a few small vacuoles, there was a small number of exfoliated epithelial cells in the tubular lumen, and the renal capsule lumen was partially narrowed (Fig. 2).

**Fig. 3:**
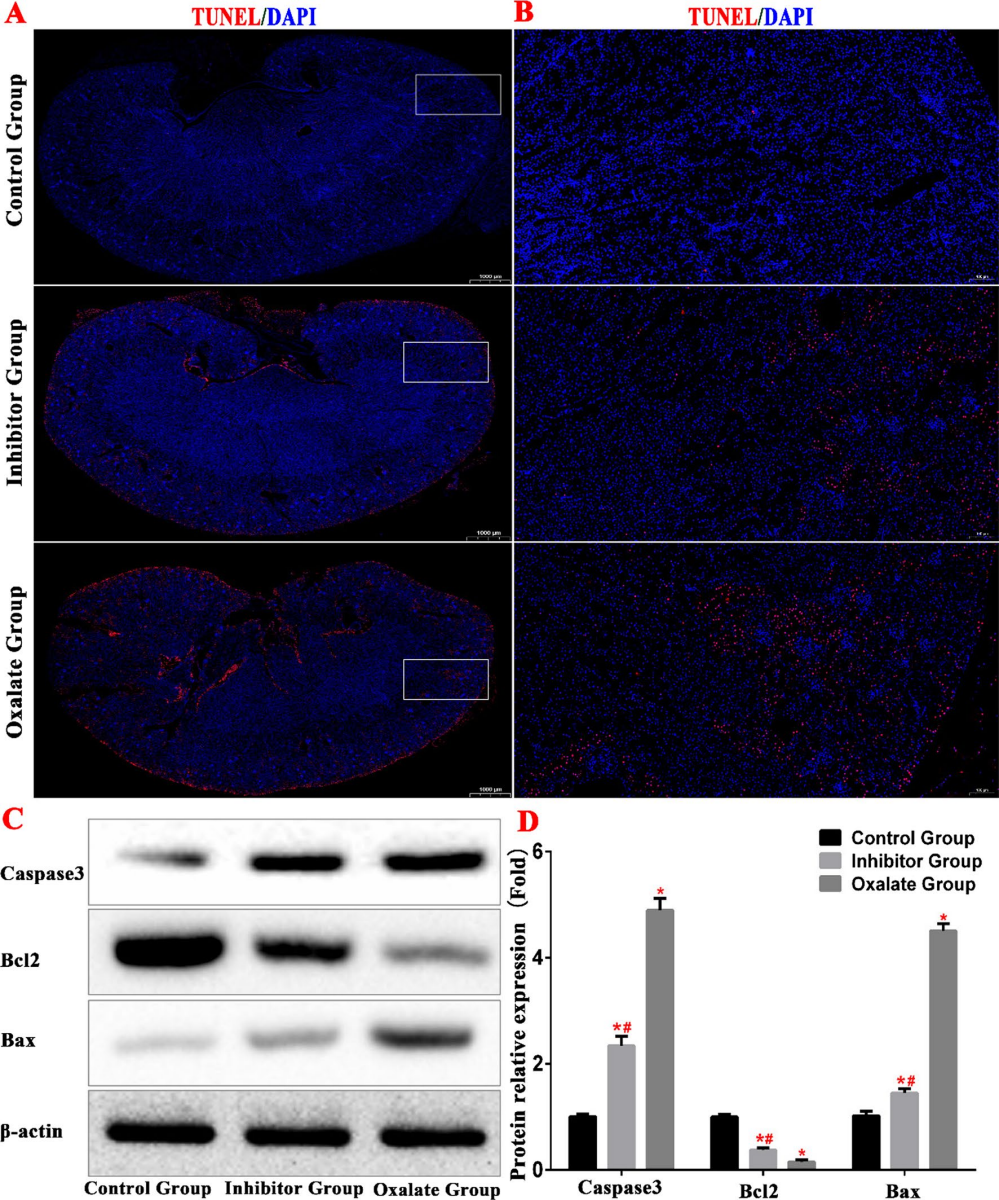
**A**, **B** TUNEL staining was used to observe the renal apoptosis of mice in each group. **C**, **D** Western blot was used to detect the expression of apoptosis-related proteins. (*^(Red)^Compared with control group, *P* < 0.01. ^#(Red)^Compared with oxalate group, *P* < 0.01)

TUNEL revealed that the apoptosis of renal tubular epithelial cells increased in the oxalate group, whereas it decreased in the inhibitor group. These results were corroborated by the western blotting analysis, which revealed that the caspase 3 and Bax protein levels increased, whereas the Bcl-2 levels decreased. This indicates that the apoptosis of renal tubular epithelial cells increased in the kidneys of the oxalate group mice (Fig. 3).

### Oxalate induces autophagy, apoptosis, and mitochondrial damage in HK-2 cells through the ERS pathway

Autophagy is a biological process that involves complex molecular mechanisms. During autophagy, double-membrane sac structures form, which wrap the unfolded proteins and damaged organelles and combine with lysosomes to form autophagosomes (Wu et al. 2018). An appropriate level of autophagy has a protective effect on cells, whereas excessive autophagy may accelerate apoptosis (Delgado and Tesfaigzi 2013). LC3 is a very important autophagy protein that has two forms: LC3-I and LC3-II. At the beginning of autophagy, LC3-I is transformed into LC3-II; therefore, an increase in the LC3-II/LC3-I ratio indicates the initiation of autophagy (Runwal et al. 2019). Beclin1 protein mediates autophagy; the level of Beclin1 is positively correlated with the level of autophagy (Lv et al. 2019). The autophagy ligand protein p62 can be anchored to a protein to be degraded, and its level decreases during autophagy. Therefore, a low p62 protein content indicates smooth autophagy downstream.

**Fig. 4:**
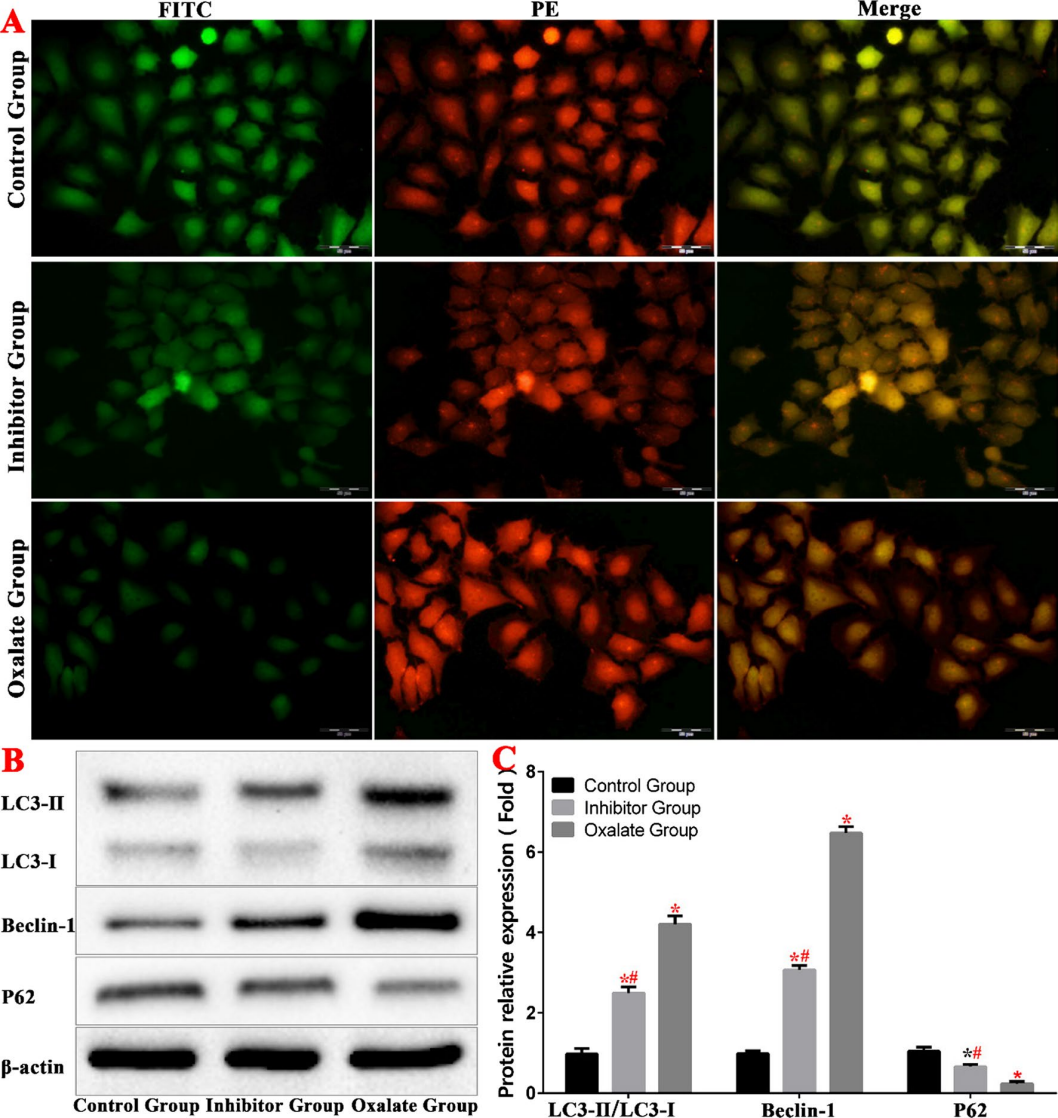
Oxalate-induced autophagy in HK-2 cells. **A** Autophagy level of each group of autophagy lentivirus. **B**, **C** Western blot was used to detect the expression of autophagy-related protein in each group.(*compared with normal Control group, *P* < 0.05.*^(Red)^Compared with control group, *P* < 0.01. ^#(Red)^Compared with oxalate group, *P* < 0.01)

To determine whether autophagy is involved in oxalate damage in HK-2 cells, we used mRFP/mCherry-GFP-LC 3 B tandem fluorescent protein to detect autophagy flow levels in each group. When autophagy is activated, the fluorescence signal attributable to GFP is quenched after the GFP enters the lysosome, which has a relatively low pH. However, the mRFP and mCherry fluorescent groups are more stable than GFP with regard to pH, and retain their fluorescence after entering the autophagy lysosome. The results showed that, compared to the control group, the number of red spots in the oxalate group increased significantly, whereas green fluorescence decreased. This indicates that the number of autophagy lysosomes in the oxalate group increased significantly, and the level of autophagy in the cells increased accordingly (Fig. 4).

Western blotting analysis revealed that in the oxalate group, the LC3-II/LC3-I ratio increased, the protein level of Beclin1 increased, and the level of P62 decreased. These results also indicated that the autophagy of tubular epithelial cells increased in the oxalate group (Fig. 4).

In the inhibitor group, the LC3-II/LC3-I ratio decreased, the protein level of Beclin1 decreased, and the level of P62 increased compared to the corresponding values in the oxalate group, indicating that autophagy decreased in the inhibitor group (Fig. 4).

**Fig. 5:**
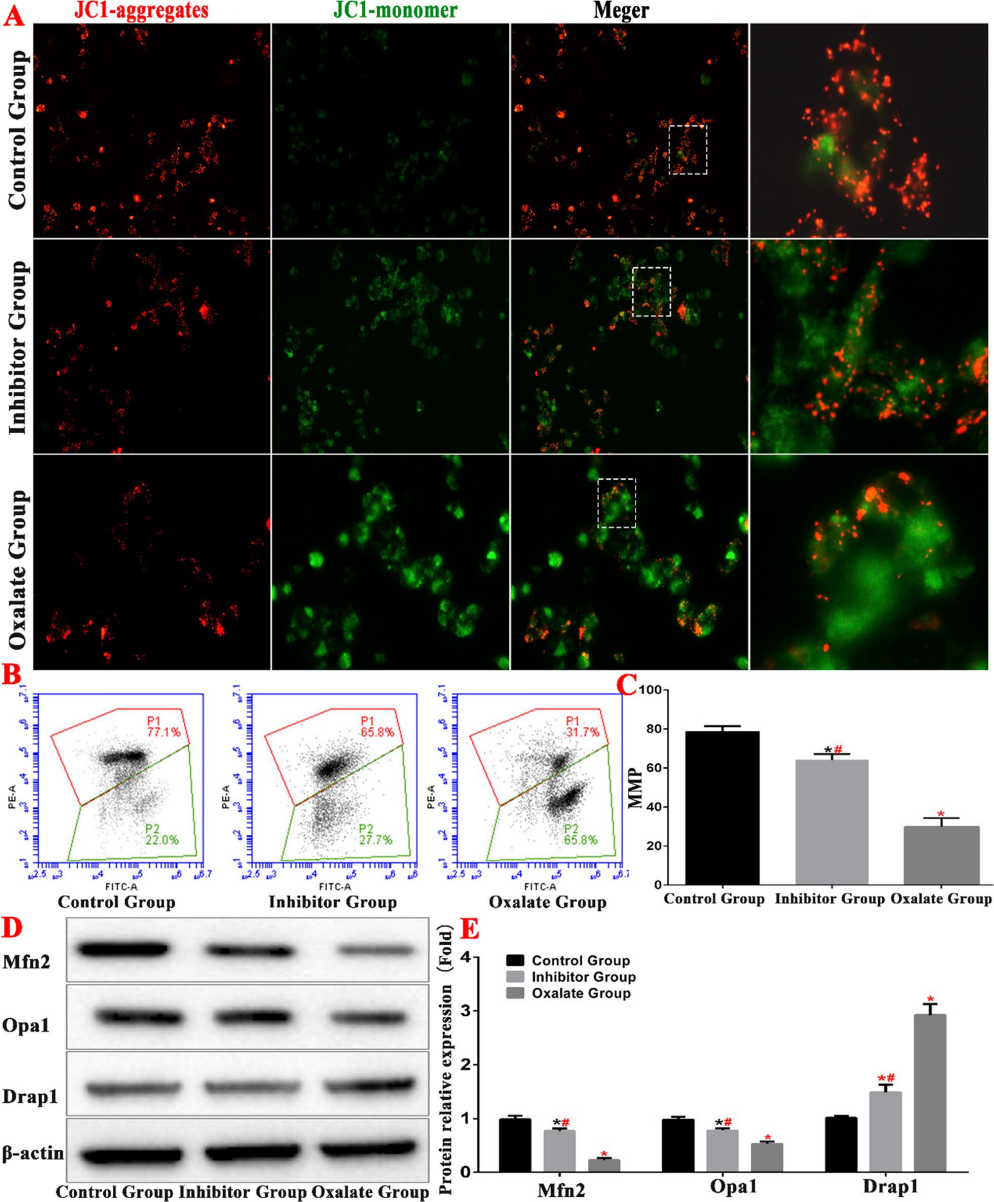
Oxalate-induced mitochondrial damage in HK-2 cells. **A** MMP detection in JC-I fluorescence groups. **B**, **C** Comparison of MMP detection in each group by flow cytometry. **D**, **E** Western blot was used to detect the expression of mitochondrial related protein in each group (*compared with normal control group, *P* < 0.05.*^(Red)^Compared with control group, *P* < 0.01. ^#(Red)^Compared with oxalate group, *P* < 0.01)

In this study, we used the JC-1 method to detect cell mitochondrial membrane potential (Fig. 5). In normal mitochondria, JC-1 accumulates in the mitochondrial matrix to form a polymer, which produces intense red fluorescence. During a decrease or loss of membrane potential, JC-1 can only exist in the cytoplasm in the form of monomers, which produce green fluorescence. In the present study, we detected mitochondrial membrane potential in the cells of each group by fluorescence staining. We found that the control group cells emitted mainly red fluorescence, whereas the cells of the oxalate group emitted mainly green fluorescence. The cells of the inhibitor group emitted a stronger red light and a weaker green light than those of the oxalate group. MMP detection by flow cytometry also showed that the mitochondrial membrane potential of the oxalate-treated cells (in the oxalate group) was significantly lower than that of the cells in the control group (*P* < 0.01). The mitochondrial membrane potential increased significantly in the inhibitor group compared to that in the oxalate groups (*P* < 0.01).

Western blotting revealed that the expression level of mitochondrial mitogen DRP1 in the oxalate group increased, the expression levels of mitochondrial fusion proteins OPA1 and Mfn2 decreased, the expression level of inhibitor group DRP1 decreased, and the expression levels of OPA1 and Mfn2 increased. These results indicate that the ERS pathway is involved in oxalate-induced mitochondrial kinetic imbalance in HK-2 cells (Fig. 5).

**Fig. 6:**
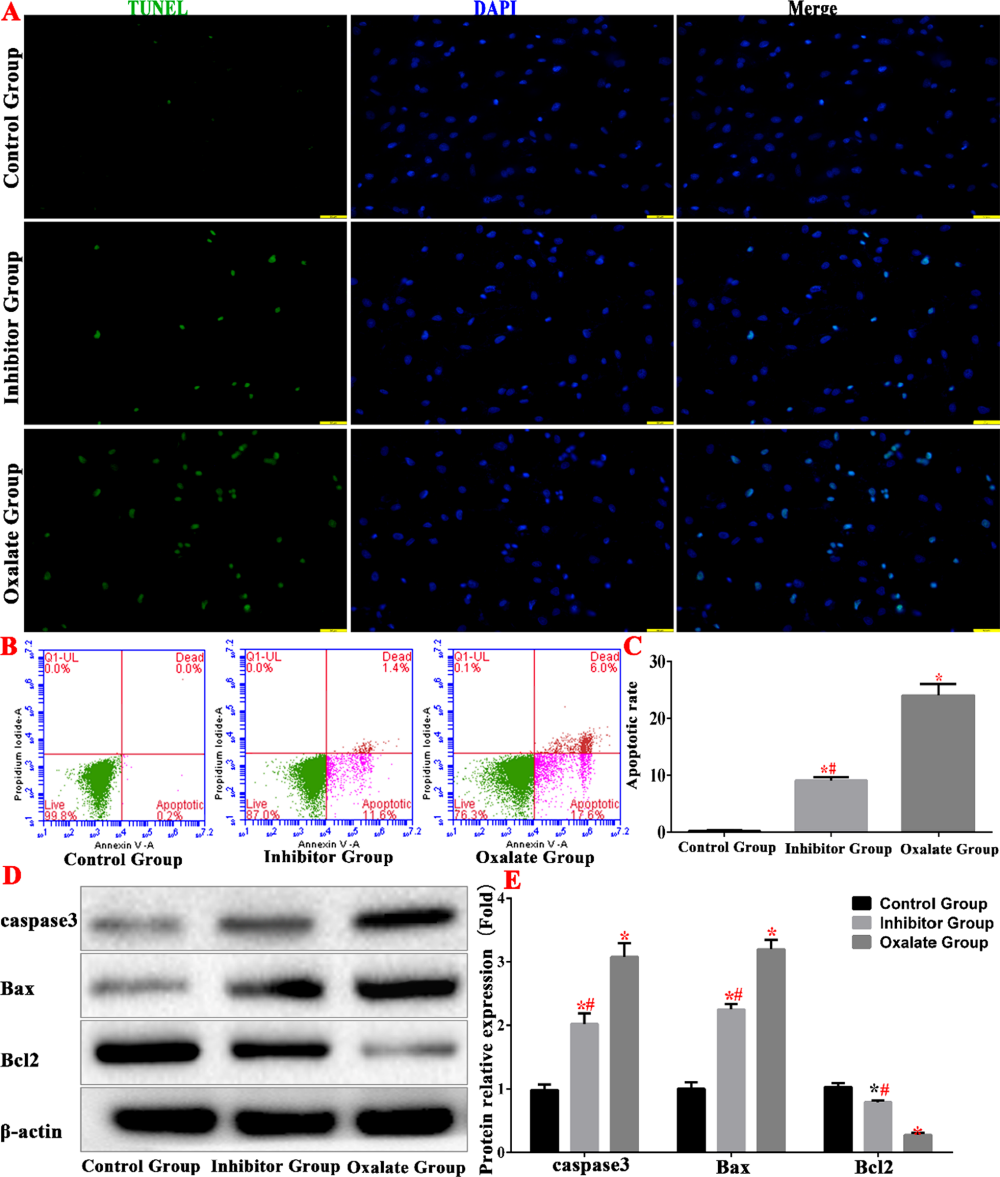
Oxalate-induced apoptosis of HK-2 cells. **A** detection of apoptosis in each groups. **B**, **C** Comparison of apoptosis detection in each group by flow cytometry. **D**, **E** Western blot was used to detect the expression of apoptosis-related proteins in each group. (*compared with normal control group, *P* < 0.05.*^(Red)^Compared with control group, *P* < 0.01. ^#(Red)^Compared with oxalate group, *P* < 0.01)

The TUNEL assay and flow cytometry results revealed that the apoptosis rate increased significantly in the oxalate group, which further verified the effect of the ERS pathway on the oxalate-induced apoptosis of renal epithelial cells. Western blotting analysis also showed that the caspase 3 and Bax levels increased in the oxalate group, whereas Bcl-2 levels decreased. In the inhibitor group, the apoptosis rate decreased, and the levels of caspase 3 and Bax proteins decreased compared with the oxalate group. The increase in Bcl-2 levels indicate that ERS inhibition also inhibited apoptosis (Fig. 6).

### Oxalate activates ERS and the NF-κB signalling pathway in renal tissue

**Fig. 7:**
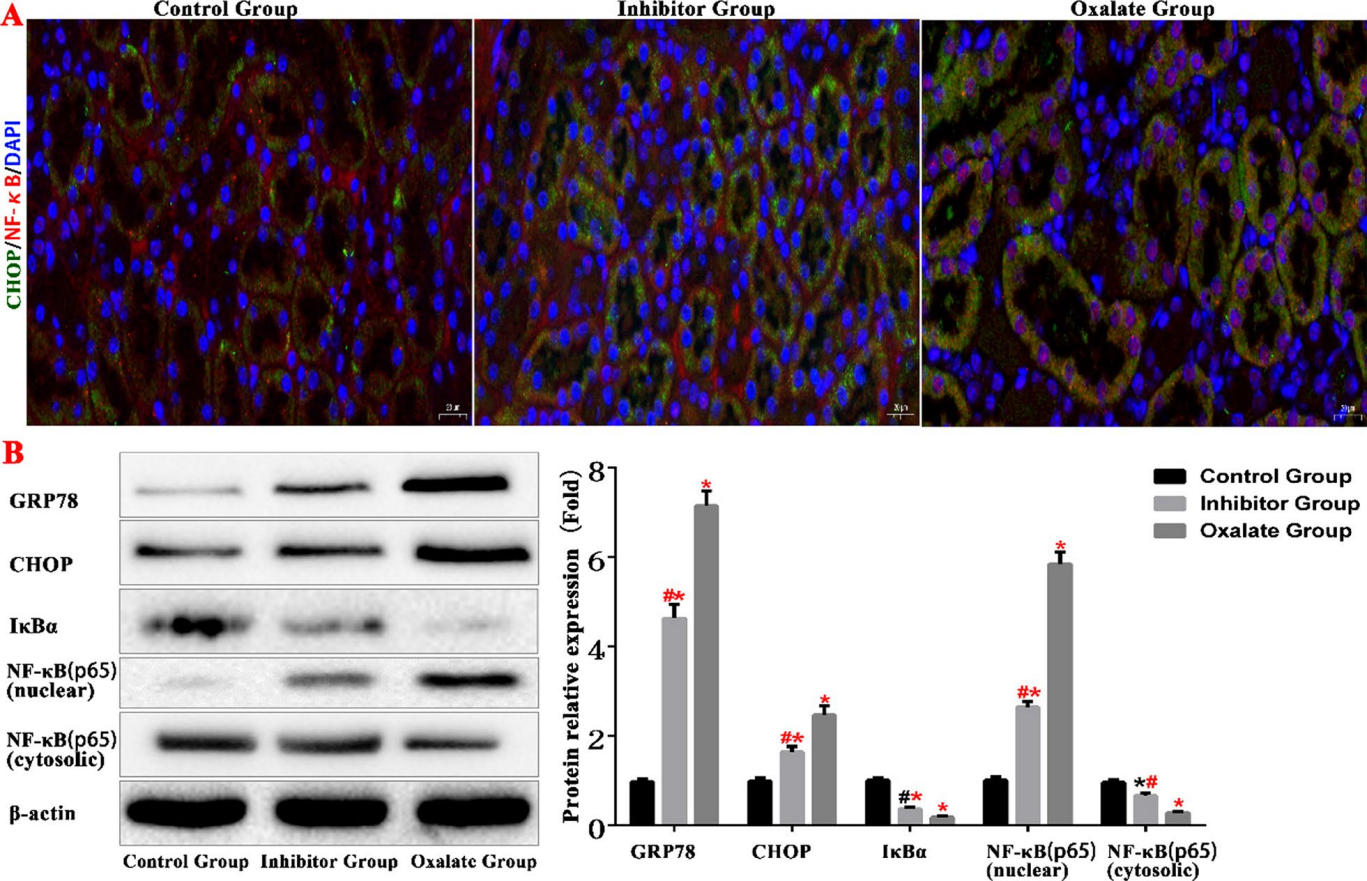
ERS and NF-κB protein expression level in kidney tissue of mice (*compared with normal Control group, *P* < 0.05.*^(Red)^Compared with Control group, *P* < 0.01. ^#^Compared with oxalate group, *P* < 0.05. #^(Red)^ Compared with oxalate group, *P* < 0.01)

To study the effects of oxalate stimulation on ERS and the NF-κB signalling pathway in kidney tissue cells, we used an immunofluorescence assay to determine CHOP and NF-κB protein expression levels in the renal tissues of the three groups. We found that the CHOP and NF-κB protein levels increased in the renal tubular epithelial cells of the oxalate group, the nuclei of which experienced a significant NF-κB protein aggregation (Fig. 7).

We used western blotting to detect differences in the levels of the ERS proteins GRP78 and CHOP, and in those of NF-κB and NF-κB inhibitor alpha (IκBα) proteins in the kidney tissues of the mice in the three groups. In the oxalate group, the levels of GRP78, CHOP, and NF-κB (nuclear) increased, whereas the levels of IκBα and NF-κB (cytosolic) decreased. These results indicate that oxalate stimulation activates ERS and the NF-κB signalling pathway in renal tissue (Fig. 7).

### Oxalate activates ERS and the NF-κB signalling pathway in HK-2 cells

**Fig. 8:**
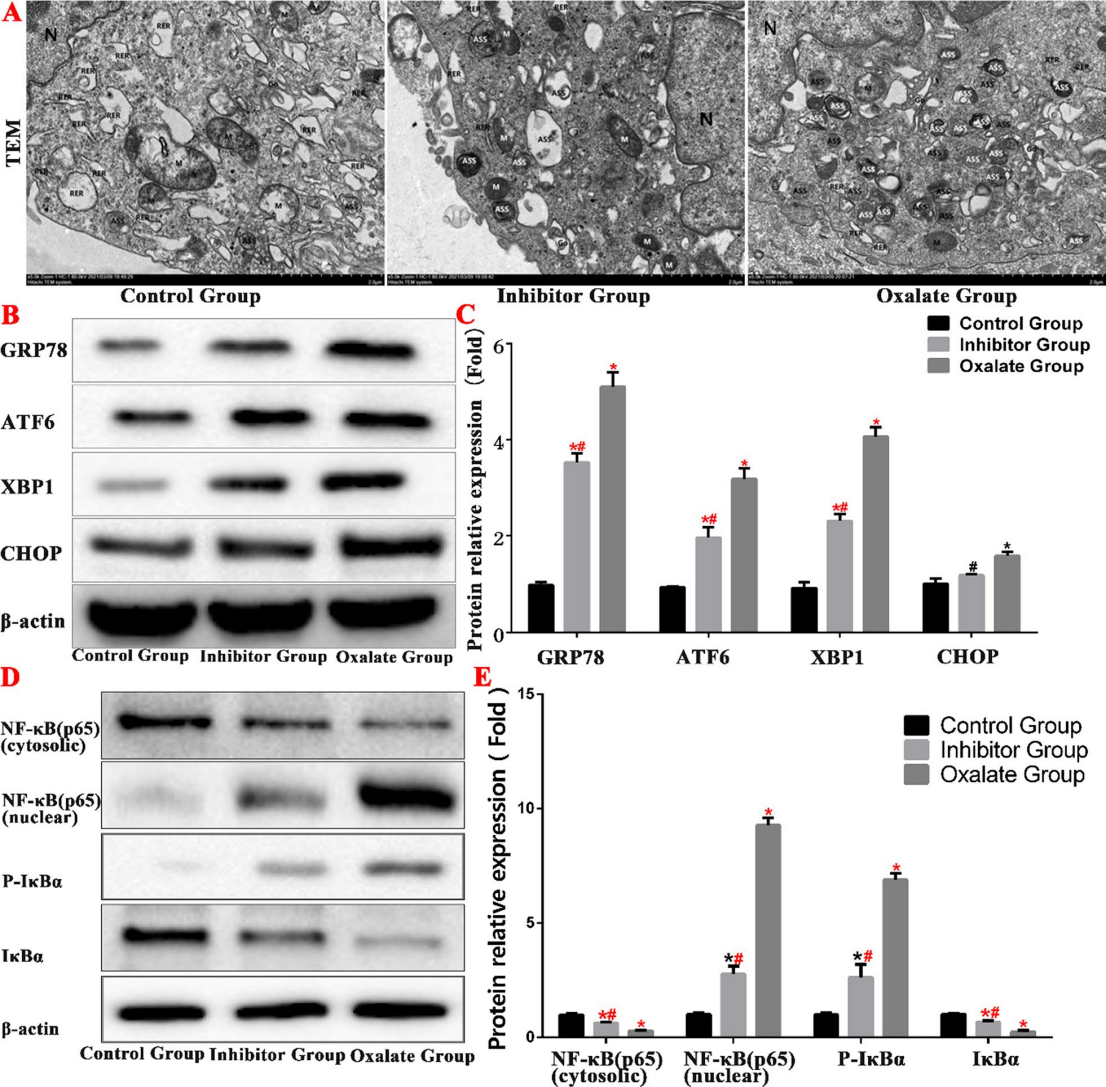
Oxalate activates the NF-κB pathway by stimulating ERS in HK-2 cells. **A** Ultrastructure observation of each group under transmission electron microscope. **B**, **C** Western blot was used to detect the expression of ERS-related proteins in each group. **D**, **E** Western blot was used to detect the expression of NF-κB pathway proteins in each group. (*compared with normal Control group, *P* < 0.05.*^(Red)^Compared with Control group, *P* < 0.01. ^#^Compared with oxalate group, *P* < 0.05. ^#(Red)^Compared with oxalate group, *P* < 0.01)

An examination of the ultrastructure of the oxalate group cells under a transmission electron microscope revealed that the morphology of the nuclei (n) was markedly irregular, the edges of the nuclear membranes were clear, the local nuclear membranes were markedly depressed, the gaps around the nuclei had widened, the chromatin in the nuclei was evenly distributed, the amount of heterochromatin had decreased, the mitochondria (m) were moderately swollen, some mitochondrial matrices had become lighter, and the cristae had blurred, diminished, and disappeared. The rough ER had expanded moderately in some cells and markedly in others, and some ERs were vacuolated. The Golgi apparatus were moderately dilated and there were numerous autophagy lysosomes in the cytoplasm. However, in the inhibitor group there were numerous swollen mitochondria, a few of the mitochondrial matrices were faint, and the cristae were blurred, which reduced the number of autophagy lysosomes in the cytoplasm. There were fewer autophagy lysosomes in the inhibitor group than that in the oxalate group (Fig. 8).

In the present study, we examined the expression of ERS-related proteins in HK-2 cells after oxalate treatment. The expression levels of GRP78, ATF6, XBP1, and CHOP proteins in the oxalate group were significantly higher than those in the normal group. This indicates that ERS was significantly higher in the oxalate group than that in the normal group. The expression levels of GRP78, ATF6, XBP1, and CHOP in the inhibition group decreased significantly compared to those in the oxalate group (Fig. 8).

We determined the expression levels of IκBα, p-IκBα, NF-κB (cytosolic), and NF-κB (nuclear) proteins by western blotting. The expression levels of NF-κB (nuclear) and p-IκBα proteins were higher in the oxalate group than those in the control group. The NF-κB signalling pathway was significantly suppressed in the inhibitor group compared to that in the oxalate group (Fig. 8).

### Oxalate activates the ROS by stimulating ERS

**Fig. 9:**
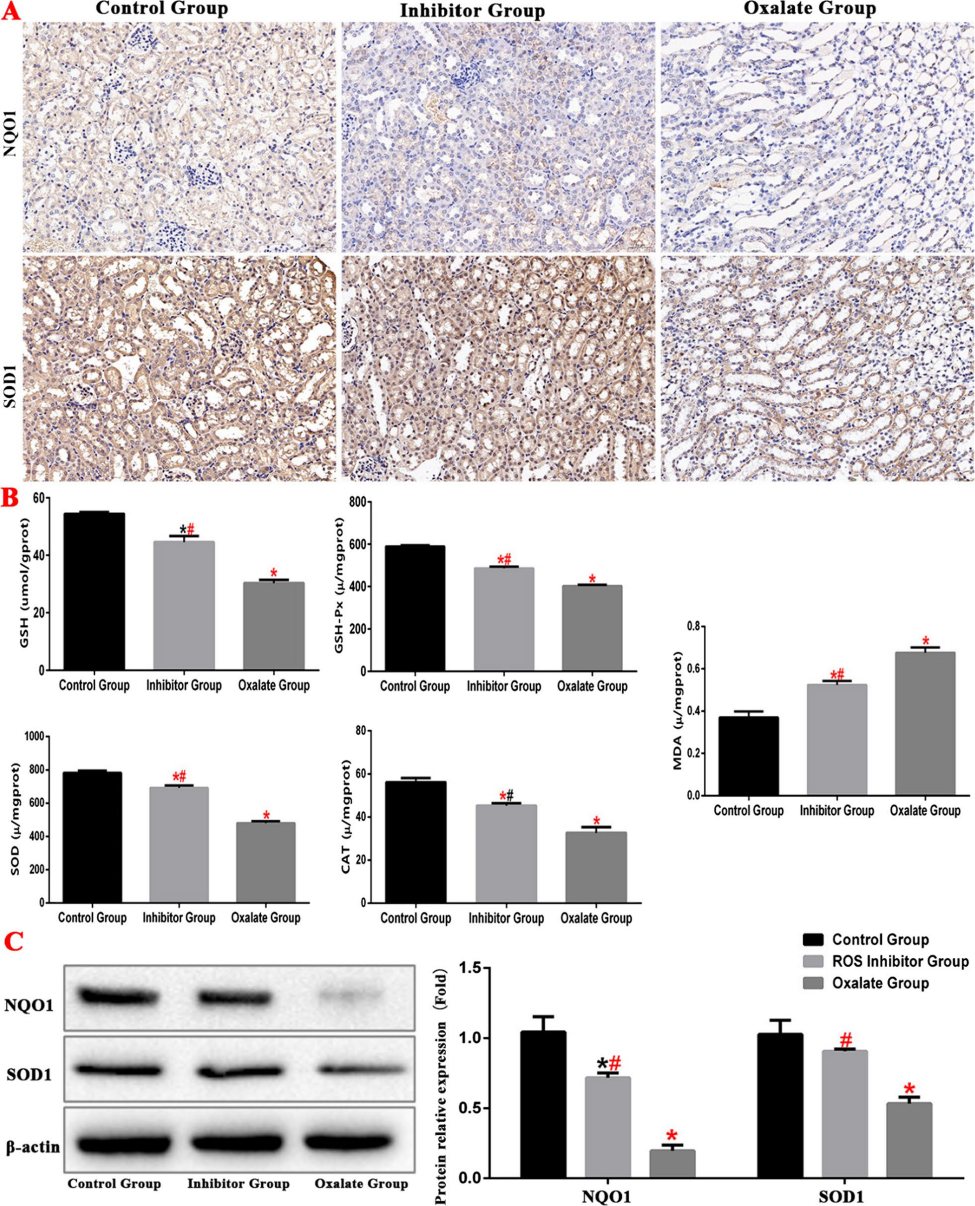
ERS and ROS protein expression level in kidney tissue of mice in each group. (*compared with normal control group, *P* < 0.05.*^(Red)^Compared with control group, *P* < 0.01. ^#^Compared with oxalate group, *P* < 0.05. ^#(Red)^Compared with oxalate group, *P* < 0.01)

Oxidative stress is the damage due to the higher pro-oxidant effect than the antioxidant effect in vivo. In this study, we detected the expression of ROS-related proteins in the renal tissues of model mice and found that in the oxalate group, the expression of NQO1 and SOD1 was downregulated, the contents of SOD, GSH, GSH-Px and CAT decreased, and the content of MDA increased, indicating an increase in ROS generation; the antioxidant damage in the inhibitor group was significantly increased (Fig. 9).

**Fig. 10:**
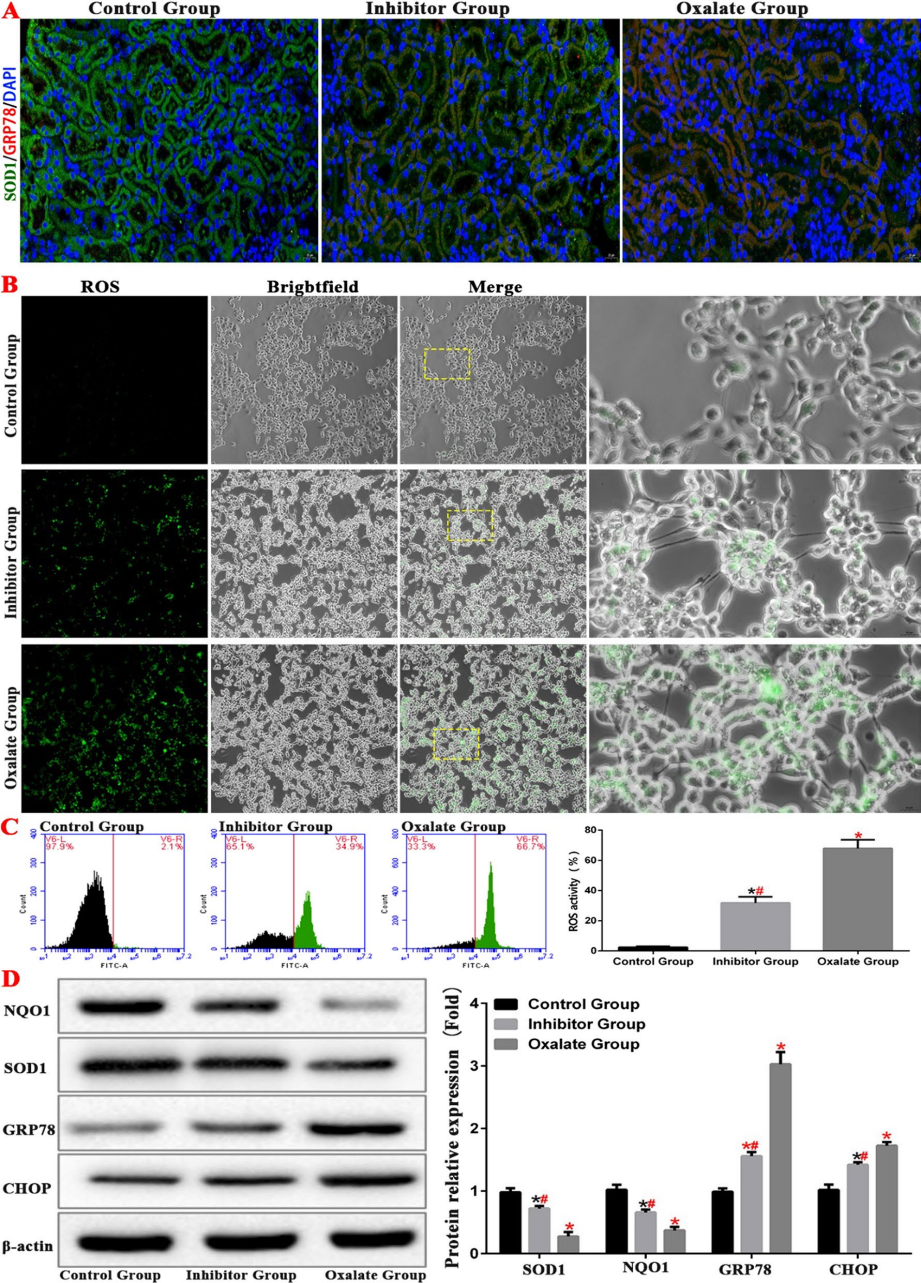
ERS inhibits ROS levels in kidney tissue of mice and HK-2 cell. **A** The expressions of GPR78 and SOD1 in the kidney of model mice were detected by immunofluorescence. **B**, **C** The level of ROS in HK-2 cell model was detected by fluorescence microscope and flow cytometry. **D** Western blot was used to detect the expression of ERS and ROS related proteins protein in HK-2 cell model. (*compared with normal control group, *P* < 0.05.*^(Red)^Compared with control group, *P* < 0.01. ^#^Compared with oxalate group, *P* < 0.05. ^#(Red)^Compared with oxalate group, *P* < 0.01)

In order to further verify the relationship between ers and oxidative stress, we tested the level of ROS and the expression of related proteins in HK-2 cells through cell experiment, the results showed that inhibition of ERS could down regulate the level of oxidative stress (Fig. 10).

### ROS induces apoptosis of HK-2 cells by activating NF-κB signaling pathway

**Fig. 11:**
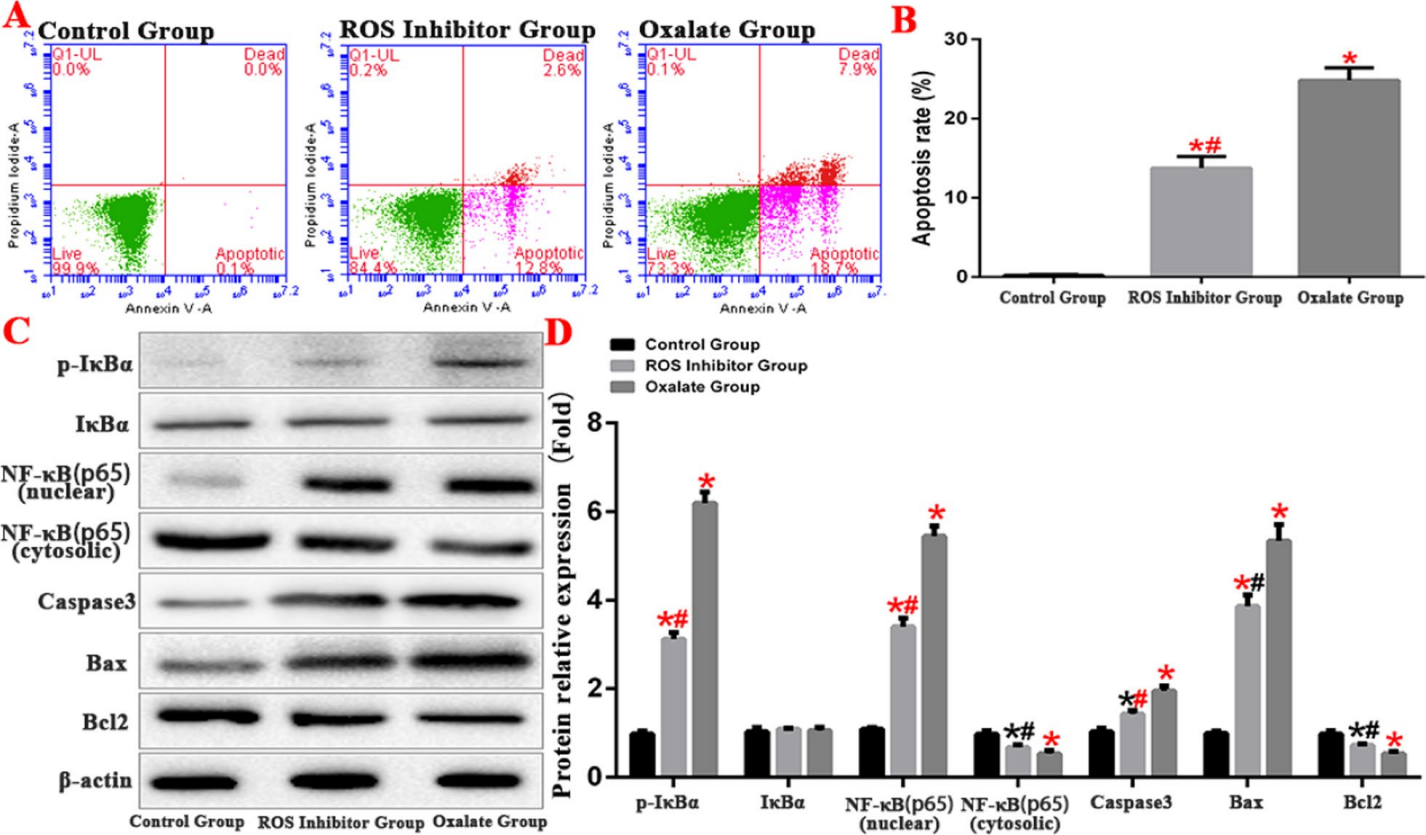
ROS induces apoptosis of HK-2 cells by activating NF-κB signaling pathway. **A**, **B** Comparison of apoptosis detection in each group by flow cytometry. **C**, **D** Western blot was used to detect the expression of apoptosis-related proteins and NF-κB protein in each group. (*compared with normal Control group, *P* < 0.05.*^(Red)^Compared with Control group, *P* < 0.01. ^#^Compared with oxalate group, *P* < 0.05. ^#(Red)^Compared with oxalate group, *P* < 0.01)

NF-κB signalling pathway is among the important regulatory pathways of ROS. In this study, we established the ROS inhibition group by treatment with the ROS inhibitor HTHQ (1-*O*-hexyl-2,3,5-trimethylhydroquinone) to reduce ROS levels in cells. The results showed that the apoptosis rate was significantly downregulated after treatment with the ROS inhibitor. The expression of NF-κB signaling pathway-related proteins was detected by western blot, and results showed that ROS promotes IκBα phosphorylation, thus further regulating the activity of the cellular NF-κB signalling pathway (Fig. 11).

### Inhibition of NF-κB pathway can inhibit the level of ERS/ROS in cells

**Fig. 12:**
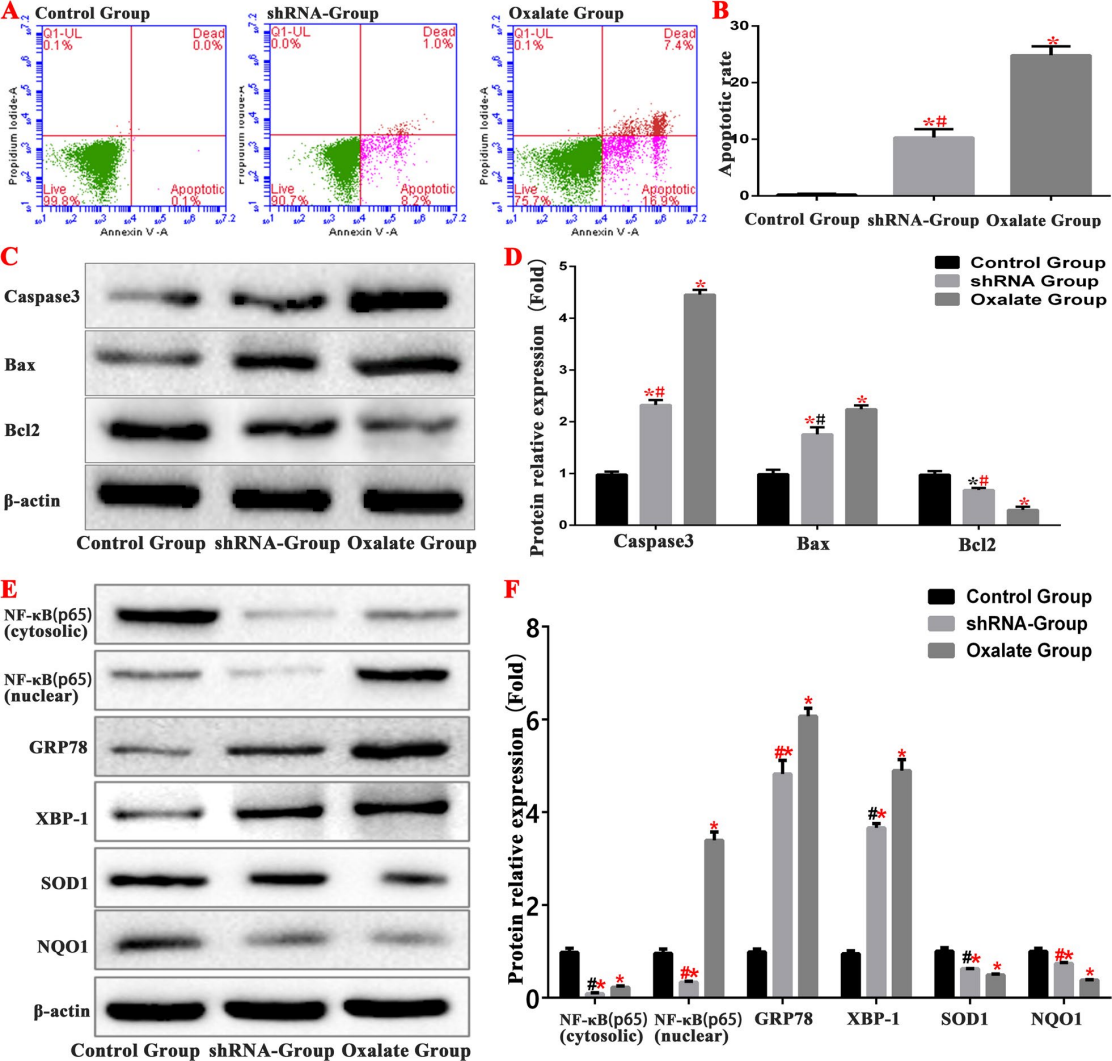
Inhibition of NF-κB signaling pathway can inhibit ERS and ROS levels in cells. **A**, **B** Comparison of apoptosis detection in each group by flow cytometry. **C**, **D** Western blot was used to detect the expression of apoptosis-related proteins and NF-κB protein in each group. **E**, **F** Western blot was used to detect the expression of ERS and ROS related proteins and NF-κB protein in each group. (*compared with normal Control group, *P* < 0.05.*^(Red)^Compared with control group, *P* < 0.01. ^#^Compared with oxalate group, *P* < 0.05. ^#(Red)^Compared with oxalate group, *P* < 0.01)

To further verify that oxalate activates the NF-κB signalling pathway in HK-2 cells either through ERS or ROS, we created an NF-κB interfering lentivirus group—the small hairpin RNA (shRNA) group—which was also stimulated by oxalate. The changes in the expression levels of ERS- and ROS-related proteins GRP78, XBP1, NQO1, SOD1 and NF-κB in the shRNA group were consistent with those in the oxalate group. However, after the downregulation of NF-κB expression by lentivirus interference, the protein levels of NF-κB (cytosolic) and NF-κB (nuclear) in the shRNA group decreased (Fig. 12).

The rate of apoptosis in the shRNA group was significantly lower than that in the oxalate group. We determined the expression levels of apoptosis-related proteins. The expression levels of caspase 3 and Bax decreased, and those of Bcl-2 increased in the shRNA group, which further confirms that oxalate induces apoptosis in HK-2 cells through activates the NF-κB signalling pathway (Fig. 12).

Interestingly, comparison of the expression of ERS- and ROS-related proteins, including GRP78, XBP1, NQO1 and SOD1, between the shRNA and oxalate groups showed that the levels of ERS and ROS were also significantly inhibited after inhibition of the NF-κB pathway (Fig. 12).

## Discussion

Kidney stones are common and frequently recur. Consequently, they are economically expensive. Therefore, prevention of their occurrence and recurrence has become one of the biggest challenges in modern urology (Jones et al. 2017). Kidney stones can be removed by shockwave lithotripsy, ureteroscopy, percutaneous nephrolithotomy, and other surgical operations (Knoll et al. 2012; El-Husseiny and Buchholz 2012). However, recurrence rates after stone removal are high and the number of new cases continues to increase (Hyams and Matlaga 2014). The formation of kidney stones is a complex process that involves the supersaturation of urolithiasis factors, the injury of renal tubular epithelial cells, adhesion, aggregation, nucleation, and the growth of crystals (Gul and Monga 2014). The damage caused by oxalic acid and calcium oxalate crystals to the renal tubular epithelial cells is one of the key factors in stone formation (Loi et al. 2021).

Glyoxylic acid is converted to oxalate by the liver; this promotes the formation of calcium oxalate kidney stones in mice (Lange et al. 2012). In this study, we established a model of oxalate kidney stones by intraperitoneally injecting glyoxylic acid into mice. Morphological comparisons revealed that the kidneys in the oxalate group increased in size and weight, the kidney tissues swelled, and the renal capsules tightened. Haematoxylin and eosin staining showed that the proximal tubules of the oxalate group changed markedly, the epithelial cells swelled, and numerous vacuoles appeared in the cells. The facial brush edge disappeared, necrotic cell fragments and interstitial oedema appeared in the tubule cavity, and the renal tubule damage grade scores were higher than those in the contact and inhibitor groups. The renal apoptosis test showed that apoptosis increased in the oxalate group, and the western blotting test showed that ERS and the NF-κB signalling pathway were over-activated. However, the addition of an ERS inhibitor markedly alleviated renal injury in the inhibitor group. The renal tissue was basically normal and apoptosis was reduced. Furthermore, ERS and the NF-κB signalling pathway were inhibited, suggesting that they play important roles in oxalate-induced renal injury.

To further investigate the role and mechanism of ERS in the oxalate-induced injury of renal tubular epithelial cells, we established a cell model by in vitro experiments. The results from both the animal model and the cell experiments confirmed that oxalate significantly activates ERS in cells and induces cell damage. Inhibiting the ERS process can significantly reduce autophagy and apoptosis, and protect mitochondrial function.

The ER is an important organelle and has the physiological function of regulating the normal steady state of cells. When cells experience nutritional deficiency, hypoxia, oxidative stress, and other factors, the functions of ER processing, modification, and protein transportation become disordered, resulting in ERS. Many studies have shown that ERS mainly affects IRE1-XBP1, PERK-eIF2α, ATF6, and IRE1, which are transmembrane ER proteins that form dimers and undergo autophosphorylation when free. Phosphorylated IRE1 splices introns in the precursor mRNA molecule of X-box binding protein 1 (XBP1) (Lin and Popko 2009). PKR-like ER kinase (PERK)/eukaryotic initiation factor 2 (eIF2) is an important pathway that regulates ERS. When cells are stimulated, it causes the overexpression of PERK, which further induces the recruitment and phosphorylation of its substrate EIF2. This causes ER abnormality and regulates the biological functions of cells. eIF2α phosphorylation inhibits protein translation and synthesis. It reduces the influx of newly formed proteins to the ER and further increases the levels of unfolded proteins (Song and Kim 2017). ATF6 is an ER receptor protein, and an important regulator of apoptosis and autophagy induced by ERS. It initiates the ERS pathway and the ERS-mediated apoptosis signalling pathway to induce apoptosis. Studies have shown that apoptosis-promoting transcription factors such as CHOP, c-JNK, and caspase 12 are activated when cells are exposed to excessive or sustained ERS (Wang et al. 2018). CHOP is a unique ERS-associated transcription factor, and is the most important marker protein in ERS-induced apoptosis (Ning et al. 2016). Under normal conditions, it is present in very low levels, mainly in the cytoplasm. However, during cell stress its expression increases, which can induce CHOP expression through the three pathways mentioned above. The overexpression of CHOP downregulates the expression of anti-apoptotic proteins BCL2, BCL-XL, and MCL-1, and upregulates the expression of BIM. This promotes the expression of BAK and BAX, leading to apoptosis (Zhou et al. 2018).

The ER is the initial site of autophagy (He et al. 2019). Autophagy is an important downstream event in ERS and is upstream of apoptosis. ERS-mediated autophagy plays a dual role in cell survival by regulating apoptosis (Zhang et al. 2019). Various causes of ERS, such as Ca^2+^ disorders, activate the unfolded protein response (UPR) pathway and autophagy by regulating the expression of UPR-related genes. This reduces the load of secreted proteins, which increases the removal of unfolded proteins, eliminating damaged organelles and protecting cells from apoptosis (Cybulsky 2017). Although autophagy is a cellular defence mechanism against dangerous stimuli, it can also participate in ERS-induced apoptosis. When ERS exceeds the intensity threshold or critical duration and UPR is insufficient to alleviate it, UPR over-activates autophagy. This results in the complete degradation of cell components or the failure of autophagy to perform its normal functions, which in turn results in apoptosis. Studies have shown that IRE1α can also trigger autophagy by activating autophagy-associated genes such as Beclin1, or by upregulating the expression of Nedd4-2 through XBP1 transcription mediated by its RNase activity (Wang et al. 2016; Cai et al. 2016). IRE1α activation can also lead to the production of phosphorylated MAPK8 and Beclin1, which are the main downstream regulators of MAPK8. Activated MAPK8 can directly phosphorylate Bcl-2, thereby destroying the interaction between Beclin1 and Bcl-2 and inducing autophagy (Rashid et al. 2015; Giacomello and Pellegrini 2016).

Mitochondria-associated ER membranes play important roles in oxidative stress, ERS, apoptosis, autophagy, dynamic changes in mitochondria, fluidity, and inflammatory reactions. Mitochondrial autophagy is specific to damaged mitochondria. It maintains the dynamic balance between mitochondrial division and fusion, and the normal function of mitochondria in cells (Sprenger and Langer 2019). However, the decrease in membrane potential and ATP synthesis during mitochondrial dysfunction results in reactive oxygen species and cell damage. Under normal circumstances, there is a dynamic balance between the fusion and division of mitochondria. However, during stimulation by diseases or harmful factors, that balance is disrupted, leading to mitochondrial dysfunction (Frank et al. 2001). Mitochondrial fusion is mainly regulated by Mfn and OPA1 genes: Mfn mediates the fusion of the mitochondrial outer membrane and OPA1 mediates the fusion of the inner membrane. Mitochondrial division is mainly regulated by DRP1. Studies have shown that the hyperactivation of DRP1 causes excessive mitochondrial division and the formation of mitochondrial fragments. It also induces oxidative stress, deficiency in energy metabolism, and the synthesis of pro-apoptotic factors Bax (Wu et al. 2017; Zhao et al. 2021).

ERS and oxidative stress are important mechanisms to maintain cell homeostasis. ERS interacts with oxidative stress, autophagy, and apoptosis through the unfolded protein response signalling pathway, an important link in determining cell fate under stress. Oxidative stress refers to the in vivo reaction of free radicals and ROS with proteins, lipids, nucleic acids, and other polymers due to damaged antioxidant mechanism, resulting in damage to the structure and function of the these substances. Aerobic oxidation and ROS are produced during cell respiration and energy metabolism. NQO1, which protects cells from oxidative stress by preventing quinone from entering the single-electron reduction process (Ross and Siegel 2021). When excessive ROS is produced in the body and ROS clearance is slowed down due to the weakening of the antioxidant capacity, the oxidation/reduction balance in the body is broken, leading to oxidative stress. ROS can cause DNA oxidative damage, lipid peroxidation, protein denaturation, or inactivation of tissues and cells and participate in intracellular signal pathway transduction (Ray et al. 2012). Aging, neurodegenerative diseases, inflammatory diseases, cancer, and other major diseases are also related to oxidative stress (Sena and Chandel 2012). There is an effective antioxidant defence system in cells. Antioxidant enzymes, such as SOD, cat, GSH and GSH PX, play an important role in eliminating ROS (Yang et al. 2012). SOD is an important free radical-scavenging enzyme in the first line of defence against oxidative damage and plays an important role in protecting cells from oxidative damage (Khan et al. 2014). The decrease in SOD activity leads to the excessive production of superoxide anion and hydrogen peroxide in the biological system, resulting in lipid peroxidation (Zhang et al. 2014). MDA is the final product of lipid peroxidation, and its level can reflect the degree of oxidative damage of tissues and cells. This study confirmed that oxalate could increase the levels of SOD, GSH, and GSH-Px in mouse kidney and reduce that of MDA. Cell experiments showed that oxalate induces renal injury was related to oxidative stress injury due to ERS.

The NF-κB pathway promotes inflammatory responses. NF-κB usually exists as a homologous or heterologous complex composed of the NF-κB/Rel family (Luo et al. 2019). At rest, cytoplasmic NF-κB combines with its inhibitor IκB to form a trimer. Therefore, IκB is an important inhibitor of the NF-κB signalling pathway and prevents nuclear translocation. When cells are stimulated by extracellular signals, IκB is reduced during synthesis or degraded by phosphorylation, releasing NF-κB for nuclear translocation. It therefore plays a role in regulating cellular function (Xu et al. 2018). In this study, both the animal model and the cell experiments showed that oxalate activates ERS/ROS and the NF-κB signalling pathway. Studies have shown that during ERS, after the separation of PERK protein and GRP78, PERK is activated by dimerization and autophosphorylation, and the downstream eukaryotic translation initiation factor 2α (EIF2α) is phosphorylated to form P-EIF2α. P-EIF2α activates NF-κB by reducing the synthesis of IκB (Wang et al. 2018; Jiang et al. 2018; Vallée et al. 2020; Ye et al. 2021b). To further verify the effect of oxalate-induced ERS activation on the activation of the NF-κB signalling pathway. Furthermore, the NF-κB signalling pathway is an important target of ROS. By ROS stimulation, IκB is phosphorylated, and its proteasome is degraded. Subsequently, the activated NF-κB is phosphorylated and translocated to the nucleus (Hou et al. 2019), which further regulates the release of related inflammatory cytokines and aggravates the inflammatory response (Lin et al. 2016). In addition, the activation of the NF-κB pathway further promotes the process of ERS and ROS generation, leading to the feedback loop of ERS, ROS, and the NF-κB pathway and aggravating damage to renal tissues.

This study showed that oxalate activates the ERS/ROS/NF-κB signalling pathway to induce renal tubular epithelial cell injury, however the related mechanism needs to be verified in human samples. Moreover, we found that HK-2 cells show apoptosis even after inhibition the NF-κB signalling pathway, indicates that this signalling pathway is not the only mechanism of ERS-induced apoptosis. Future studies should investigate other related mechanisms.

## Conclusion

This study showed that oxalate activates the ERS/ROS/NF-κB signalling pathway to induce renal tubular epithelial cell injury. Our findings can facilitate the pharmacological intervention of oxalate-induced renal tubular epithelial cell injury.

## Data Availability

All data generated or analysed in the present study are included in this published article and its supplementary information files. For more information, please contact the authors of this article.

## Abbreviations

DAPI: 4′,6-Diamidino-2-phenylindole
ER: Endoplasmic reticulum
ERS: Endoplasmic reticulum stress
ROS: Reactive oxygen species
MMP: Mitochondrial membrane potential
NF-κB: Nuclear factor kappa-light-chain-enhancer of activated B cells
PBS: Phosphate-buffered saline
TUNEL: Terminal deoxynucleotidyl transferase-mediated nick end labelling
UPR: Unfolded protein response
